# TRIM4-mediated ubiquitination of NSP2 restricts porcine reproductive and respiratory syndrome virus proliferation

**DOI:** 10.1186/s12917-022-03309-1

**Published:** 2022-05-30

**Authors:** Mengmeng Zhao, Huiyang Sha, Hang Zhang, Ruining Wang

**Affiliations:** 1 School of Life Science and Engineering, Foshan University, No. 33, Guangyun Road, Nanhai District, Foshan, 528000 China; 2 College of Veterinary Medicine, Henan University of Animal Husbandry and Economy, Zhengzhou, 450046 People’s Republic of China

**Keywords:** PRRSV, NSP2, Ubiquitination, Degradation, TRIM4

## Abstract

Porcine reproductive and respiratory syndrome (PRRS) is a highly contagious and virulent infectious disease caused by the porcine reproductive and respiratory syndrome virus (PRRSV), which has substantial economic losses in the pig industry worldwide, and PRRSV attenuated vaccines and inactivated vaccines do have limitations in immune protection. The discovery of new antiviral targets has become a new research field. The proteomic studies have shown that the PRRSV NSP2 protein interacts with tripartite motif protein 4 (TRIM4), but it was still unknown whether TRIM4 regulates PRRSV infections. In this study, the *TRIM4* gene from Marc-145 cells was cloned, and it was proved that TRIM4 overexpression inhibits PRRSV replication, whereas TRIM4 small-interfering-RNA knockdown resulted in increased virus titers. Mechanism investigation indicated that TRIM4 inhibits PRRSV replication through ubiquitination and degradation of the NSP2 protein. Protease inhibitor MG132 (carbobenzoxy-Leu-Leu-leucinal) attenuated the TRIM4-driven degradation of NSP2. Taken together, TRIM4 impairs PRRSV proliferation via ubiquitination and degradation of NSP2.

**Supplementary Information:**

The online version contains supplementary material available at 10.1186/s12917-022-03309-1.

## Introduction

Porcine reproductive and respiratory syndrome (PRRS) is a highly contagious and virulent infectious disease caused by the porcine reproductive and respiratory syndrome virus (PRRSV), which has caused huge economic losses in the pig industry worldwide. With respect to immune protection, attenuated and inactivated PRRSV vaccines have limited efficacy [1]. PRRSV is an enveloped, positive-sense, single-stranded RNA virus. It has a 15 kb genome that encodes 10 open reading frames (ORF1a, ORF1b, ORF2a, ORF2b, ORF3, ORF4, ORF5, ORF5a, ORF6, and ORF7). ORF1a and ORF1b are further auto-proteolytically cleaved into different nonstructural proteins such as NSP1α, NSP1β, NSP2, NSP3, NSP4, NSP5, NSP6, NSP7α, NSP7β, NSP8, NSP9, NSP10, NSP11, NSP12 [2].

Viral NSP2 plays an important role in the regulation of various cellular functions, including regulating the antiviral immune responses, inhibition of interferon promoter gene 15, and interferon regulatory factor 3 [3, 4]. NSP2 interacts with many host proteins such as DEAD-box RNA helicase 18 [5], interleukin enhancer-binding factor 2 [6], and the cellular protein 14–3-3epsilon to modulate virus replication [7]. NSP2 regulates PRRSV infection and nuclear transcription factor-κB activity [8]. The whole NSP2 mainly includes a cysteine protease domain, a central hypervariable domain, a transmembrane domain, and a tail domain. Among them, the hypervariable domain plays a significant regulatory role in PRRSV replication; cysteine protease domains have trans and cis cleavage activity, and multiple immune epitopes on these domains mediate the host immune response [9].

The proteomics studies suggested that PRRSV NSP2 associated with the tripartite motif protein 4 (TRIM4) [10]; TRIM4 contains the RING, B-Box, Coil-Coil, and SPRY domain. Human TRIM4 regulates the interferon expression by activating retinoic acid-inducible gene I (RIG-I) and modulates hydrogen peroxide-induced cell death [11, 12]. Regarding the regulation of PRRSV replication by TRIM protein, transcriptomics experiments showed that the mRNA levels of TRIM39, TRIM41, and TRIM62 increased, and TRIM21 decreased during PRRSV infection [13]; however, the effects of TRIM4 protein on PRRSV replication and infection are still unknown. The result of our study showed that the titer of PRRSV was significantly inhibited by TRIM4 overexpression; TRIM4 small interfering RNA (siRNA) had the opposite effect against the PRRSV titer. Mechanistic studies showed that TRIM4 ubiquitinated modified NSP2 and down-regulated NSP2 expression. This study should provide a theoretical basis for further elucidating the persistent infection, immune escape, and replication mechanism of PRRSV.

## Materials and methods

### Cells and viruses

HEK-293 cells (American Type Culture Collection, Manassas, VA, # CRL-1573) and Marc-145 cells (American Type Culture Collection, Manassas, VA, # CRL-12231), an African green monkey kidney cell line which is extensively used in PRRSV research because it is permissive to infection, were grown in Dulbecco’s Modified Eagle’s Medium (DMEM) (Sigma-Aldrich, St. Louis, MO, # D6429) supplemented with 10% fetal bovine serum (Sijiqing, ZhejiangTianhang Biotechnology Co. Ltd., China, # 11011–8611), 100 U/mL of penicillin, and 100 μg/mL of streptomycin at 37 °C in a humidified atmosphere containing 5% CO_2_. The type II PRRSV BJ-4 strain (GenBank accession no. AF331831) was used for the experiments in these studies.

### Antibodies and reagents

Antibodies were as follows: anti-β-actin (Solarbio, Beijing, China, # K200058M, WB(1:1000)), anti-glyceraldehyde-3-phosphate dehydrogenase (GAPDH) (Beyotime, Shanghai, China, # AF0006, WB(1:1000)), anti-FLAG (Bioss antibodies, Beijing, China, # bs-0879R, WB(1:1000)), anti-HA (Abcepta, Beijing, China, # AM1008A, WB(1:1000)), anti-Ubiquitin(Ub) (Santa Cruz Biotechnology, Dallas, TX, # sc-8017, WB(1:1000)), anti-TRIM4 polyclonal antibody (ABclonal, Wuhan, China, # A15922, WB(1:1000)). Secondary antibodies were horseradish peroxide-conjugated rabbit anti-mouse IgG antibody (Abclonal, Wuhan, China, # WH166568, WB (1:10000)) and mouse anti-rabbit IgG antibody (Beyotime, # A0208, WB (1:1000)). The protease inhibitor MG132 (Sigma-Aldrich, M7449) was used in the study.

### RNA extraction

Total-RNA samples were extracted from Marc-145 cells by means of TRIzol (Beyotime, # R0016). RNA was stored in 40 μL of diethylpyrocarbonate (DEPC) water at − 80 °C until reverse transcription.

### Reverse transcription

The Hiscript III 1st Strand cDNA Synthesis Kit (+gDNA wiper) (Vazyme, Nanjing, China, # R312) was used for reverse transcription. The reaction mixture had a total volume of 20 μL and consisted of 11 μL of RNase-free double-distilled H_2_O (ddH_2_O), 4 μL of the 5× gDNA wiper Mix, 2 μL of the 10× RT Mix, 1 μL of the HiScript III Enzyme Mix, 1 μL of random hexamers (100 μM), and 1 μg of RNA. The reaction conditions were 37 °C for 15 min followed by 85 °C for 15 s. The complementary DNA (cDNA) was purified and then stored at − 20 °C until polymerase chain reaction (PCR) and quantitative real-time PCR (qRT-PCR).

### TRIM4 cloning

Primers TRIM4-F and TRIM4-R were used to amplify the whole *TRIM4* gene (GenBank Accession no. XM_015134414) from Marc-145 cell cDNA, and the primer sequences are shown in Table 1. The volume of the PCR mixture was 20 μL, including 10 μL of the 2× Phanta Master Mix (Vazyme, # DC401), 2 μL of cDNA, 1 μL of upstream and downstream primers (TRIM4-F, TRIM4-R, 20 μM), and 6 μL of ddH_2_O. The reaction conditions were 95 °C for 5 min, followed by 40 cycles of 95 °C for 1 min, 53 °C for 30 s, and 72 °C for 2 min. After the reaction was finished, the PCR products were subjected to 1% agarose gel electrophoresis at 150 V for 30 min. After that, the PCR products were separated with the FastPure Gel DNA Extraction Mini Kit (Vazyme, # DC301). The purified PCR products and p3XFLAG-CMV-7.1 vector (Sigma-Aldrich) were double-digested and ligated using the ClonExpress Ultra One Step Cloning Kit (Vazyme, # C115). The Mix & Go! *E. coli* Transformation Kit (Zymo Research, Irvine, CA, # T3001) and ZymoPURE Plasmid Midiprep Kit (Zymo Research, # D4200) were employed for transformation and plasmid extraction. Three clones were sent to Sangon Biotech Co., Ltd. (Shanghai, China) for sequencing.

**Table 1:** Primers used in this study

Primer	Sequence
TRIM4- F	ATCGGGATCCATGGAAGCTGAGGA
TRIM4- R	ATCGGAATTCTTTCCTGTCAGTCAC
qGAPDH-F	CTGCCGCCTGGAGAAACCT
qGAPDH-R	GCTGTAGCCAAATTCATTGTCG
qN-F	AAACCAGTCCAGAGGCAAGG
qN-R	GCAAACTAAACTCCACAGTGTAA
qTRIM4-F	AGAAGTGTTGACCAGGAGTGA
qTRIM4-R	GAAGACGAGTTTGGGATGA
Si-TRIM4	AUUCTCUUGAAGCUGUTT
Si-NC	UUCUCCGAACGUGUCACGUTT

### Plasmid transfection

The plasmids were transfected by means of the Lipofectamine 3000 Transfection Reagent (Invitrogen, Carlsbad, CA, # L3000001). The ratio of a plasmid to Lipofectamine 3000 was 1:2. The plasmid and Lipofectamine 3000 were diluted in Opti-MEM separately, mixed gently, and kept at room temperature for 15 min, and then the mixture was added to the medium of cultured cells.

### TRIM4 RNA interference depletion

The siRNA against TRIM4 was designed and synthesized by GenePharma Co., Ltd. (Suzhou, China), and its sequence is presented in Table 1. TRIM4 siRNA (60 nM, si-TRIM4) or control siRNA(60 nM, si-NC) and the Lipofectamine RNAiMAX Transfection Reagent (Invitrogen, # 13778030) were diluted in Opti-MEM, mixed gently, and kept at room temperature for 15 min, and then the mixture was added to the medium of cultured cells.

### Infection

PRRSV was diluted at a multiplicity of infection (MOI) of 1 with DMEM, and Marc-145 cells were inoculated with PRRSV at 37 °C for 1 h. Then, the cell supernatant was replaced with DMEM supplemented with 2% of fetal bovine serum.

### Quantitative real-time PCR

The mRNA level of the PRRSV genomic copy number was determined by qRT-PCR, which was carried out after cDNA synthesis. The volume of the qRT-PCR mixture was 10 μL, including 0.5 μL of upstream and downstream primers (qN-F, qN-R, 20 μM), 2.0 μL of cDNA, 5 μL of the BeyoFast SYBR Green qPCR Mix (Beyotime, # D7265), and 2.0 μL of ddH_2_O. As PCR primers, we used PRRSV N primers to amplify sequences within the 99- to 319-bp region of the *N* gene. The reaction conditions were 95 °C for 5 min, followed by 40 cycles of 95 °C for 60 s and 60 °C for 30 s (on a 7500 Fast Real-time PCR system; Applied Biosystems, Foster City, CA). The qRT-PCR primer sequences are shown in Table 1.

### Western blotting and immunoprecipitation (IP)

Western blotting and IP were performed as described previously [14–25]. In brief, cells were lysed using RIPA lysis buffer (Beyotime, #P0013B) containing 1 mg/mL protease inhibitor cocktail (Roche, Mannheim, Germany, #11873580001) for 30 min at 4 °C. These cell lysates were centrifuged for 30 min at 12,000 *g* at 4 °C.

For western blotting, the supernatants were collected and mixed with 5× sodium dodecyl sulfate-polyacrylamide gel electrophoresis (SDS-PAGE) sample loading buffer (Beyotime, #P0015). After heating for 10 min at 95 °C, BCA protein assay kit (Beyotime, #P0011) was used to quantify the protein samples, and then 30 μg proteins were loaded. The proteins were separated by a 12% SDS-PAGE separating gel (Beyotime, #P0459S) and transferred to polyvinylidene difluoride (PVDF) membranes (Bio-Rad, Hercules, CA, USA, #162–0177) in 1× Tris/Glycine Buffer (Bio-Rad, #161–0734) at 70 V for 60 min. The PVDF membranes were blocked with 5% skimmed milk (Beyotime, #P0216) at 25 °C for 1 h and incubated with diluted primary antibodies at 4 °C for 16 h. After three washes with 1× Tris-buffered saline (TBS) for 10 min each, the membranes were incubated with HRP-conjugated secondary antibodies in antibody dilution buffer (1:10,000) at 25 °C for 1 h. After three washes with 1× TBS (for 10 min each), the blots were incubated with Clarity Western ECL Substrate (Bio-Rad, #170–5060) for 5 min and developed using HyBlot CL Autoradiography Film (Denville Scientific Inc., South Plainfield, NJ, #E3018) in a dark room.

For immunoprecipitation: approximately 10% of the lysate supernatant was used as an input control and the remaining lysate was incubated overnight with EZview™ Red ANTI-FLAG® M2 affinity gel (#M2426; Sigma-Aldrich) at 4 °C. Thereafter, the beads were washed three times with lysis buffer and analyzed by western blotting using the indicated antibodies.

Gray scale analysis: Following western blotting, we determined the gray scale values of the target and internal reference bands using Image J software (https://imagej.nih.gov/ij), with relative ratio values being obtained by dividing target band values by that of the internal reference.

### Virus titers

The virus in the culture supernatant was characterized by means of the 50% tissue culture infectious dose (TCID_50_) in Marc-145 cells in a 96-well plate. Harvested cell samples were serially diluted with serum-free DMEM in a range from 10^1^ to 10^10^. The diluted samples were added to wells of 96-well plates and incubated at 37 °C for 1 h. Thereafter, the supernatant was withdrawn, cells were washed three times with PBS, and 100 uL of DMEM containing 2% FBS was added to the wells. For each dilution, we assessed eight replicates. Following infection, observations were performed over a 7-day period, during which time; we recorded the number of CPE. TCID_50_ values were calculated using the Reed–Muench method.

### Statistical analyses

All experiments were biologically repeated three times, and the data are displayed as the mean ± standard deviation of three independent assays. Paired Student’s *t* test was performed in GraphPad Prism 5.0 (GraphPad Software, San Diego, CA) to analyze the data. Data with *p* < 0.05 was considered statistically significant.

## Results

### TRIM4 inhibits PRRSV replication

**Fig. 1:**
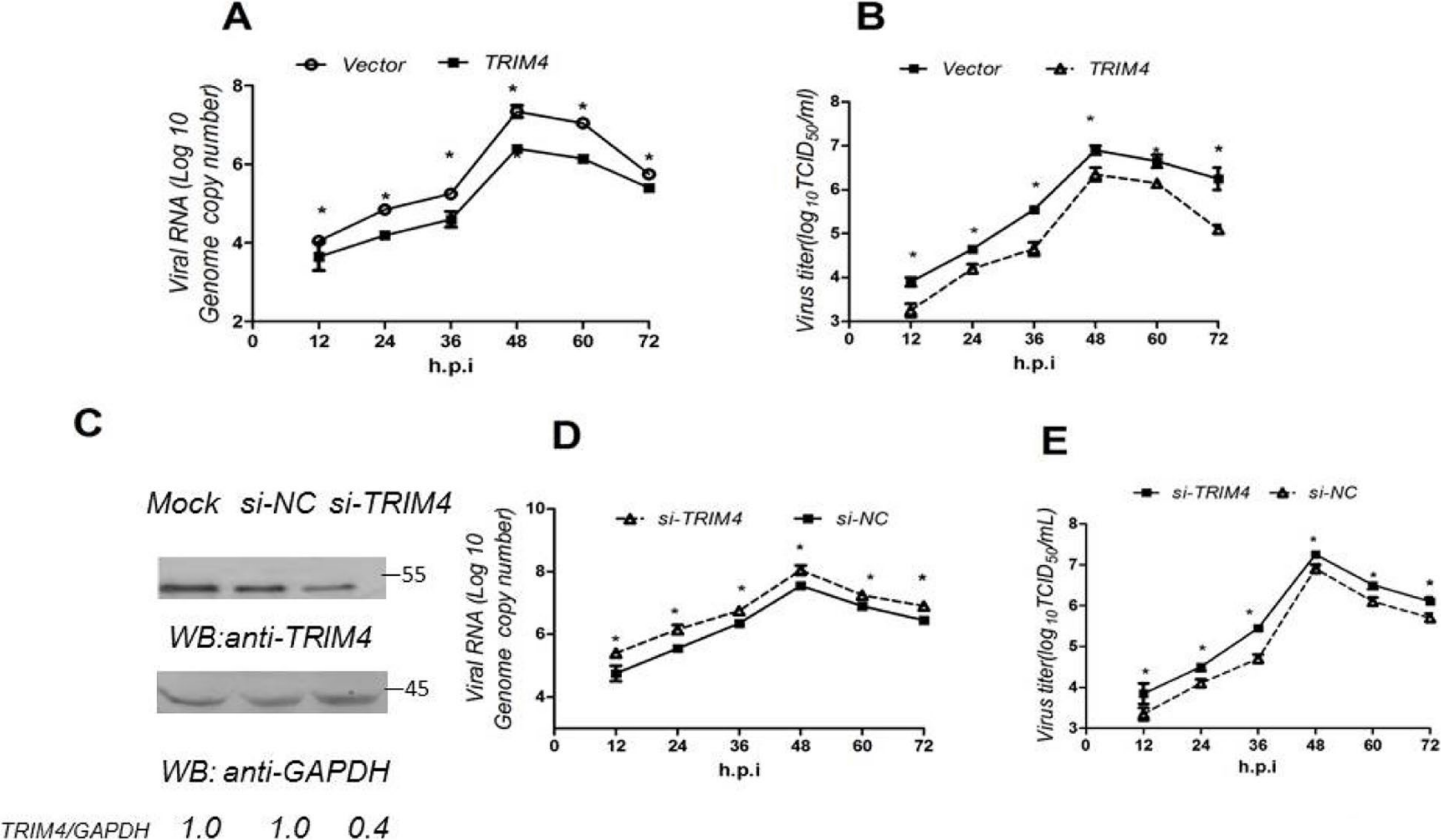
TRIM4 inhibits PRRSV replication. **A** and **B** TRIM4 overexpression inhibited PRRSV replication. FLAG-TRIM4 (0.5 μg) or p3XFLAG-CMV-7.1 vector (0.5 μg) was transfected into Marc-145 cells at over 70% confluence grown in a 24-well plate for 24 h, and PRRSV at an MOI of 1.0 was inoculated into the cells for different time points (12, 24, 36, 48, 60, and 72 h); the virus titer was determined by means of TCID_50_ (**A**), and the mRNA expression level of the PRRSV genome copy number was estimated by qRT-PCR (**B**). **C**–**E** The si-TRIM4 promotes PRRSV replication. The si-TRIM4 (60 nM) or 60 nM si-NC (60 nM) was transfected into Marc-145 cells at over 70% confluence grown in a 24-well plate for 24 h, and the TRIM4 protein level was quantified by western blotting (**C**). The si-TRIM4 (60 nM) or 60 nM si-NC (60 nM) was transfected into Marc-145 cells at over 70% confluence grown in a 24-well plate for 24 h, and PRRSV at an MOI of 1.0 was inoculated into the cells for different time points (12, 24, 36, 48, 60, and 72 h). The virus titer was characterized by TCID_50_ (**D**). The difference in the mRNA expression of PRRSV genome copy number was investigated by qRT-PCR (**E**). *P < 0.05, **P < 0.01

To verify the effect of TRIM4 on PRRSV replication, TRIM4 was overexpressed in Marc-145 cells, and then the cells were infected with PRRSV. After the designated time points (12, 24, 36, 48, 60, and 72 h), we determined the inhibitory effect of TRIM4 on PRRSV replication by the TCID_50_ and qRT-PCR methods. The results showed that TRIM4 significantly reduced the PRRSV titer (Fig. 1A) and PRRSV genome copy number (Fig. 1B). Then 60 nM si-TRIM4 or 60 nM si-NC was transfected into Marc-145 cells to verify this inhibitory effect. TRIM4 knockdown efficiency was determined by western blotting, and the results indicated that the protein level of TRIM4 decreased by 60% (based on the densitometry) (Fig. 1C). After the si-TRIM4 transfection, the cells were infected with PRRSV; after the designated time points (12, 24, 36, 48, 60, and 72 h); we evaluated the effect of si-TRIM4 on PRRSV replication by the TCID_50_ and qRT-PCR methods. The data revealed that si-TRIM4 significantly increased the PRRSV titer judging by TCID_50_ (Fig. 1D), and si-TRIM4 also significantly increased the PRRSV genome copy number (Fig. 1E). Thus, these data indicate that TRIM4 is a restriction factor against PRRSV.

To assess the inhibitory effect of TRIM4 on PRRSV replication, we also infected cells with different doses of virus. The results revealed that TRIM4 could still effectively inhibit virus replication (Fig. S1).

### TRIM4 inhibits NSP2 through ubiquitination

**Fig. 2:**
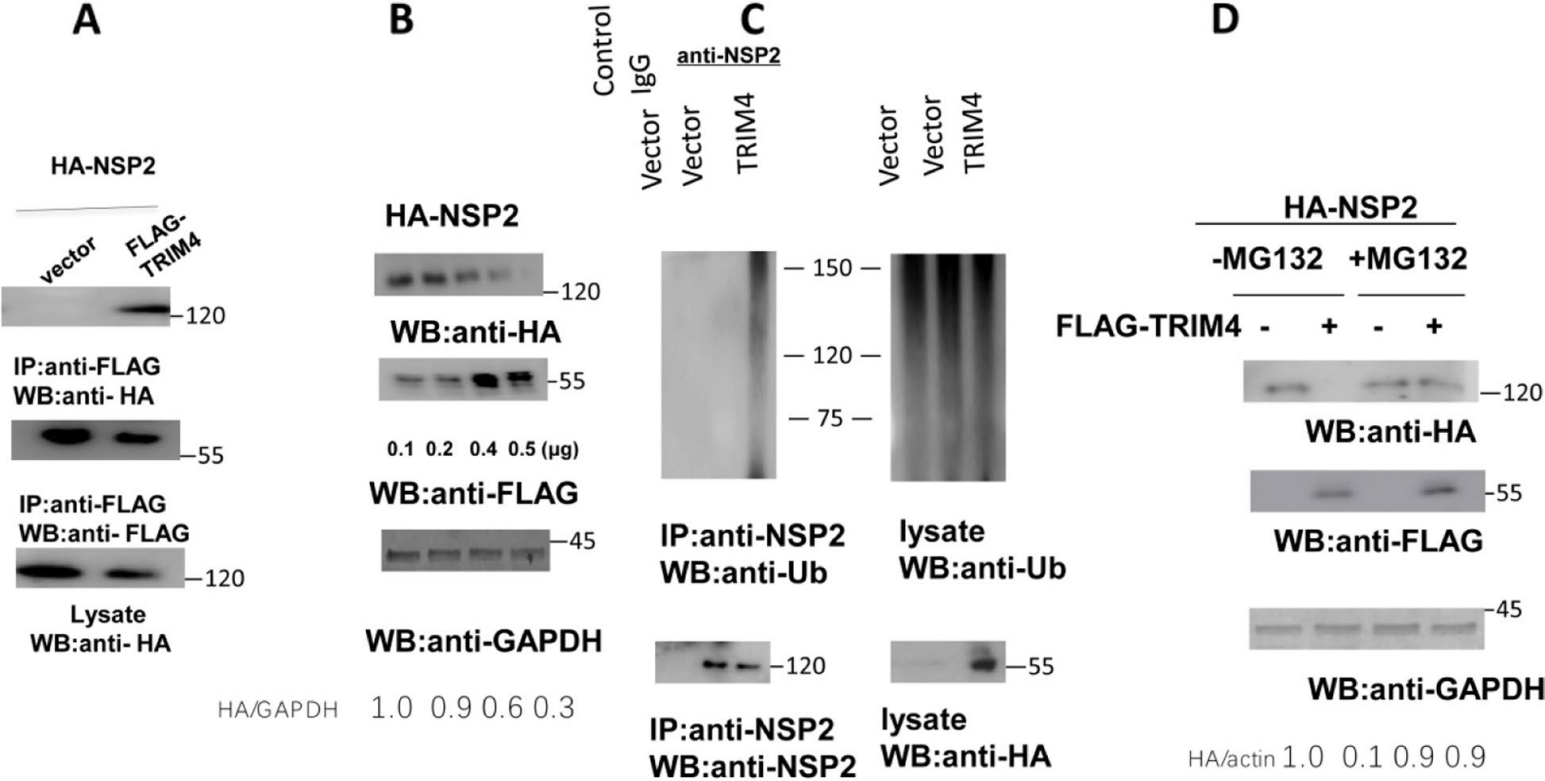
TRIM4 ubiquitinates and degrades the NSP2 protein. **A** TRIM4 interacts with NSP2. FLAG-TRIM4 (1.25 μg) and HA-NSP2 (1.25 μg) were cotransfected into HEK-293 cells at over 70% confluences grown in a 6-well plate. After 48 h, the cells were lysed with RIPA buffer and an anti-FLAG antibody was performed for an immunoprecipitation assay. Western blotting was carried out with the indicated antibodies. **B** TRIM4 degrades NSP2 expression. FLAG-TRIM4 (0.1, 0.2, 0.4, and 0.5 μg) and 0.5 μg of HA-NSP2 were transfected into HEK-293 cells at over 70% confluence grown in a 24-well plate. After 48 h, the cells were lysed with RIPA buffer, and western blotting was performed with the indicated antibody. **C** TRIM4 ubiquitinates NSP2. HA-TRIM4 (1.25 μg) and vector (1.25 μg) were co-transfected into Marc-145 cells grown in 6-well plates. After 48 h, the cells were infected with 1 MOI PRRSV, and after a further 48 h, the cells were lysed with RIPA buffer and an anti-NSP2 antibody was used for an immunoprecipitation assay. Western blotting was carried out using the indicated antibodies. **D** The effect of MG132 on the degradation of NSP2. FLAG-TRIM4 (0.5 μg) and the HA-NSP2 (0.5 μg) were cotransfected into HEK-293 cells at over 70% confluence grown in a 24-well plate, After 24 h, MG132 (5 μM) or dimethyl sulfoxide was added for 12 h. Cells were lysed with RIPA buffer, and western blotting was subjected with the indicated antibodies

The interaction between TRIM4 and NSP2 has been previously shown by proteomics [10], but the interaction is not well defined. We further validated the interaction between TRIM4 and NSP2. FLAG-tagged TRIM4 and HA-tagged NSP2 were cotransfected into HEK293 cells. The CO-IP results confirmed that TRIM4 associated with NSP2 (Fig. 2A). As TRIM4 contains an E3 ligase domain, it may regulate the target protein via ubiquitination, and we hypothesized that TRIM4 might inhibit PRRSV replication by the ubiquitination and degradation of PRRSV NSP2. To investigate this hypothesis, we cotransfected HA-tagged NSP2 and FLAG-tagged TRIM4 into HEK-293 cells for 48 h and assessed the expression of NSP2 by western blotting. The results showed that after the cotransfection, with the increasing dose of TRIM4, the expression of NSP2 decreased gradually (Fig. 2B). Next, we co-transfected the HA-tagged TRIM4 into Marc-145 cells, and after 24 h, cells were infected with 1 MOI PRRSV. Following a 48-h incubation, we used IP to quantify the levels of NSP2 ubiquitination. The results indicated that the ubiquitination level of NSP2 increased after TRIM4 was added (Fig. 2C). To explore whether the NSP2 undergoes proteasomal degradation, the protease inhibitor MG132 was added, whereas dimethyl sulfoxide was added instead in the control group. The data revealed that TRIM4 lost its ability to degrade NSP2 after MG132 was added (Fig. 2D). Taken together, combined data indicate that TRIM4 mediates ubiquitination of NSP2, which leads to NSP2 degradation and viral inhibition.

## Discussions

Innate immunity is the first line of defence against invasion by a foreign microorganism. The host recognizes foreign microorganisms by means of pattern recognition receptors. Endogenous immune factors inhibit viral replication by interacting with host factors or viral proteins. Some TRIM proteins have been reported to have antiviral effects, e.g., TRIM32 and TRIM41 [26, 27]. Our experimental results show that TRIM4 inhibits PRRSV replication through NSP2 ubiquitination, and thus TRIM4 can act as a viral restriction factor against PRRSV.

NSP2 plays a vital role in PRRSV replication. For example, NSP2 is related to cellular tropism, viral replication, antiviral immunity, and interferon production [28]. One report suggests that human TRIM4 interacts with RIG-I and targets it for K63-linked polyubiquitination [11], but whether monkey TRIM4 associates with RIG-I to activate interferon production is unknown, this may be another mechanism by which TRIM4 inhibits PRRSV replication. This phenomenon should be investigated in future research.

TRIM21 [29], TRIM22 [30], TRIM25 [31] and TRIM59 [32] have been reported to inhibit PRRSV replication, but the mechanism is unknown. TRIM22 and TRIM25 do not ubiquitinate and degrade PRRSV N protein, and N protein impairs TRIM25-mediated RIG-I ubiquitination to suppress interferon-beta production. Maybe different C-terminal domains of TRIM protein lead to different immune characteristics.

Some TRIM proteins use similar mechanisms to regulate viral replication. For example, TRIM52 interacts with and ubiquitinates Japanese Encephalitis Virus NS2A, thereby degrading viral NS2A [33], with similar mechanisms being reported for TRIM32 and influenza virus PB1 protein [27], and TRIM22 [34] and TRIM41 and influenza virus N protein [26]. Furthermore, TRIM4 has been shown to interact with SARS-CoV-2 M protein [35], which may be closely associated with susceptibility in the human population. In this study, we found that TRIM4 interacts directly with NSP2. It is reported that human TRIM4 participates in the antiviral reaction by targeting RIG-I in response to viral infection via an indirect interaction, and maintains the function of TRIM4 together.

We also cloned swine TRIM4 from porcine alveolar macrophages. Sequence analysis revealed that the nucleotide and amino acid homologies between pig and monkey were 80.7 and 70.6%, respectively, and thus we speculate that these proteins may differ functionally.

Results indicate that TRIM4 does not increase with PRRSV infection and interferon stimulation (Supplementary material Fig. S2), and it is not an interferon-stimulated gene (ISG). We speculate that TRIM4 and other TRIM proteins cooperate in the regulation of PRRSV replication. Therefore, further research should be performed in the future. TRIM4 regulation may offer a new perspective on the regulation of PRRS immune responses and serve as a reference for other medical treatments.

In summary, TRIM4 inhibits PRRSV replication via the ubiquitination of the NSP2 protein and provides intrinsic cellular defence against PRRSV infection. Upregulation of TRIM4 may activate the host immunity that suppresses viral replication. TRIM4 is a host defence factor that inhibits PRRSV replication.

## Conclusions

TRIM4-mediated ubiquitination of NSP2 limits porcine reproductive and respiratory syndrome virus infection.

## Supplementary Information

**Additional file 1.**

## Data Availability

The datasets generated and/or analysed during the current study are available in the GenBank repository (Accession no. XM_015134414 and AF331831).
