# Supplementary material for: TRIM4-mediated ubiquitination of NSP2 restricts porcine reproductive and respiratory syndrome virus proliferation
Source: BMC Vet Res. 2022 May 30;18:208. doi: 10.1186/s12917-022-03309-1 (PMC9149334; doi:10.1186/s12917-022-03309-1)
Supplement: Supplementary file 1 — Additional file 1. [file 12917_2022_3309_MOESM1_ESM.pdf]

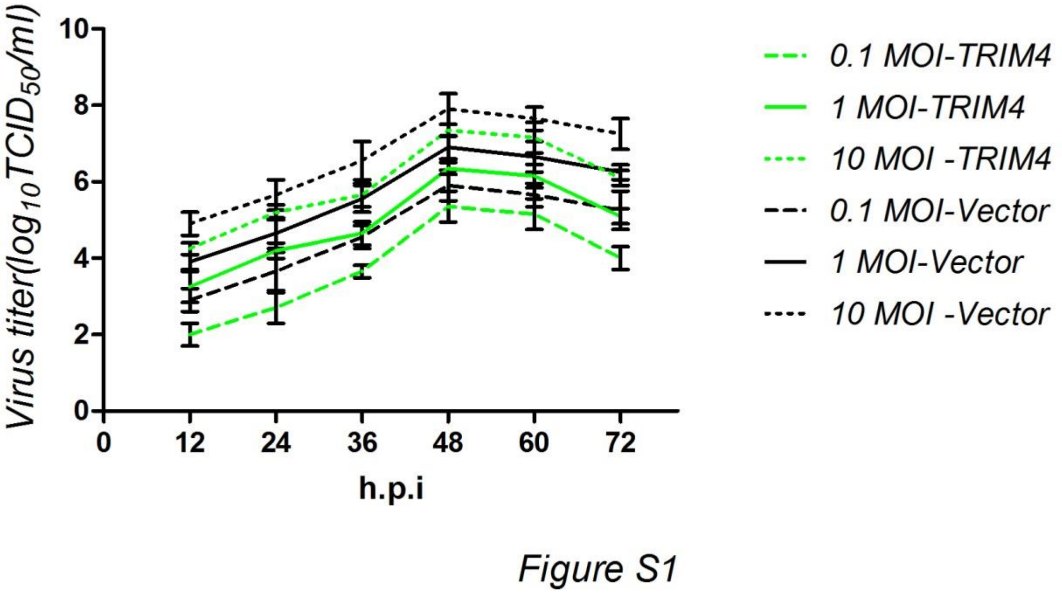

Figure S1. The effects of TRIM4 on the replication of PRRSV administered at different MOIs.

TRIM4 overexpression inhibited PRRSV replication. FLAG-TRIM4 (0.5 μg) or p3XFLAG-CMV-7.1 vector (0.5 μg) was transfected into Marc-145 cells grown in 24-well plates for 24 h at a confluence of more than 70%, and PRRSV at MOIs of 0.1, 1.0, and 10 were used to inoculated cells at different time points (12, 24, 36, 48, 60, and 72 h). The virus titer was determined by means of $TCID_{50}$.

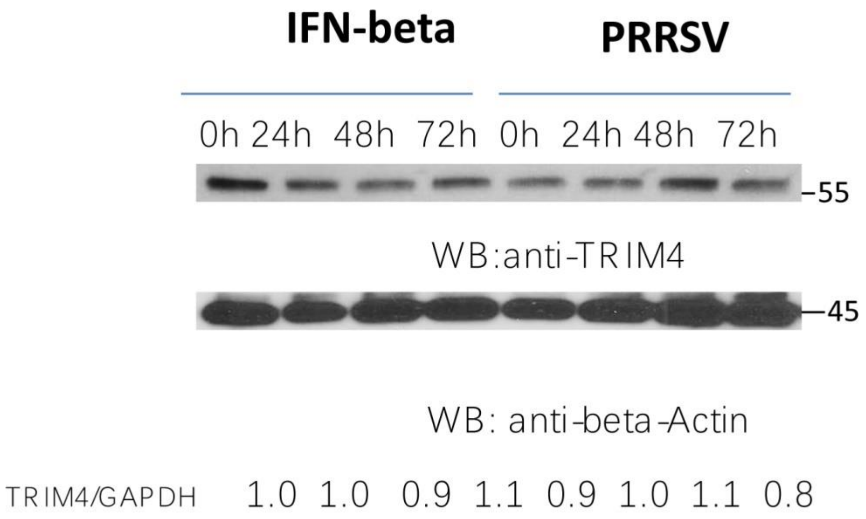

Figure S2

Figure S2. The effects of PRRSV and Interferon-beta on TRIM4 expression.

1 MOI PRRSV and 1000 IU/mL interferon-beta were used to treat PAMs, and at different time points (24, 48, and 72 h), cells were collected and subjected to western blotting using the indicated antibodies.

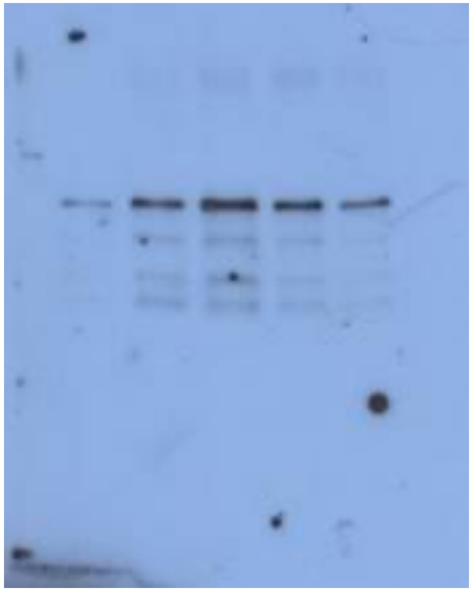

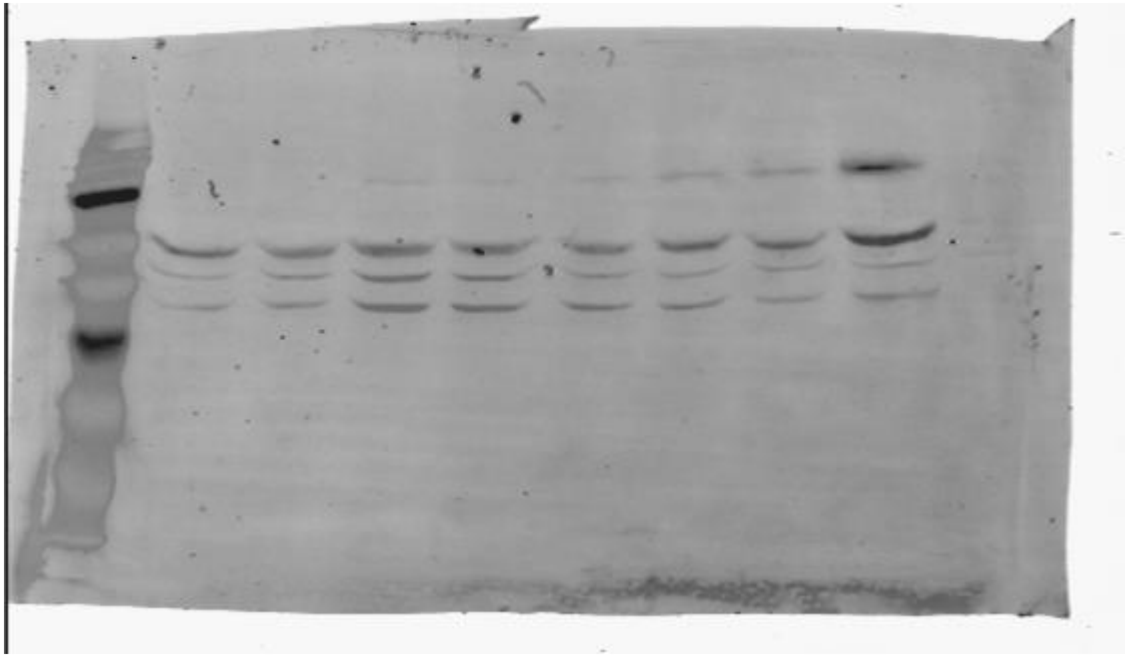

1C-GAPDH

1C-TRIM4

2A-HA

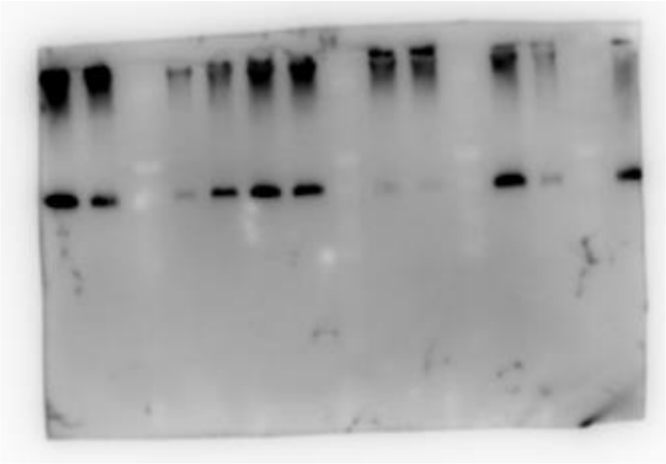

2A-FLAg

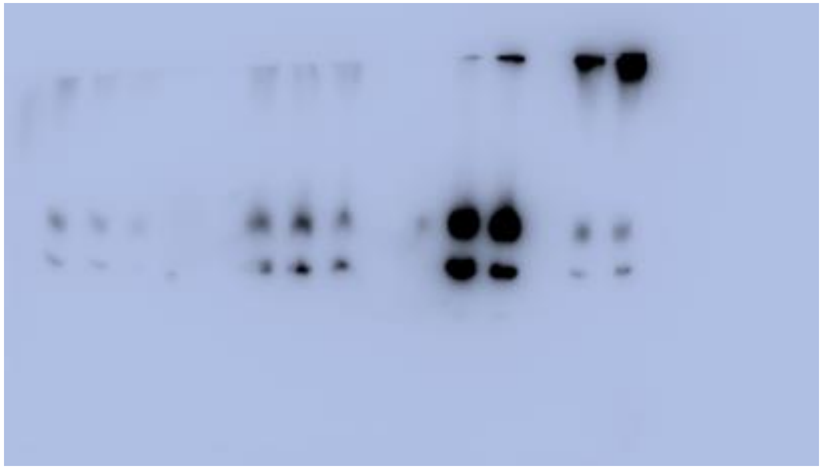

2B-HA 2B-FLAG

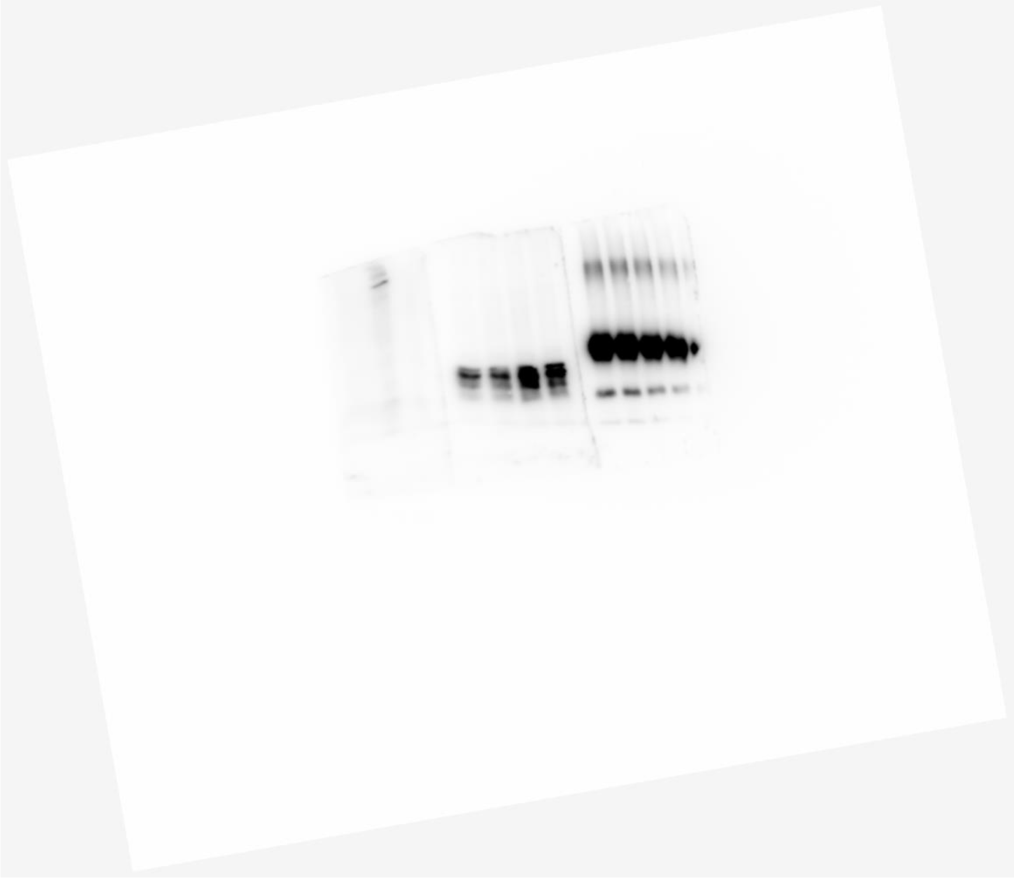

2B-GAPDH

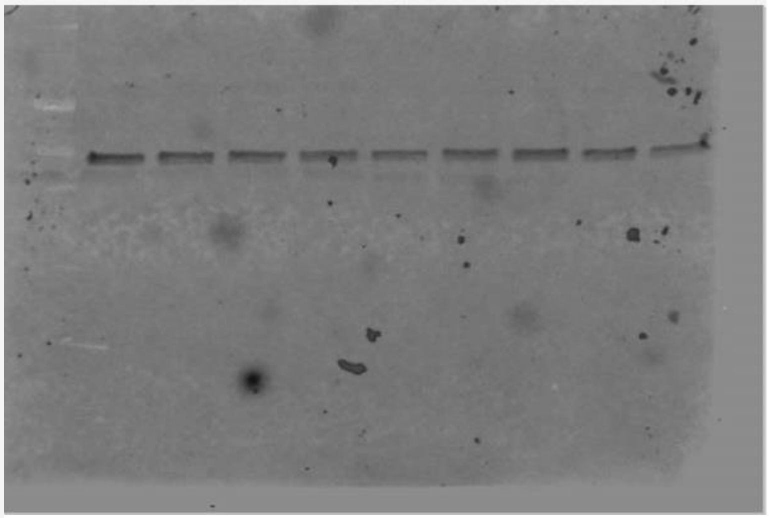

2C-UB

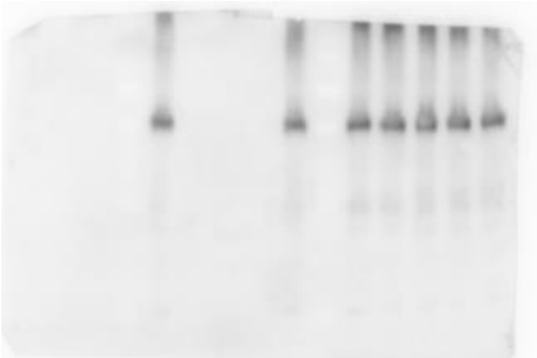

2C-UB

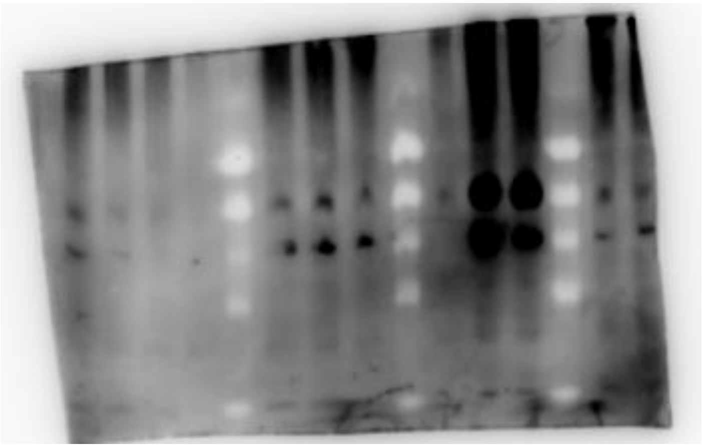

NSP2

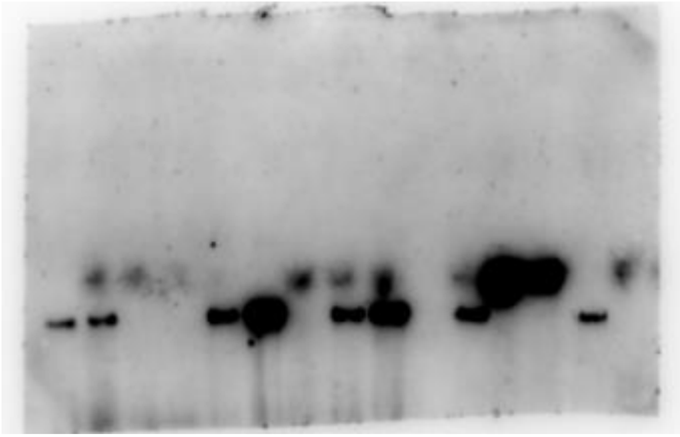

2C-HA

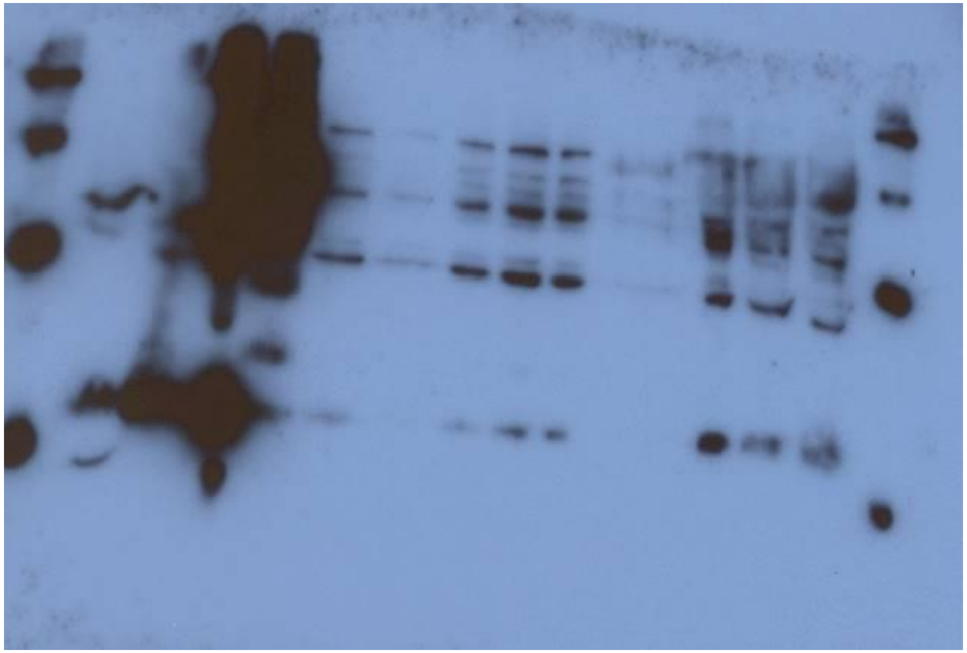

2D-GAPDH

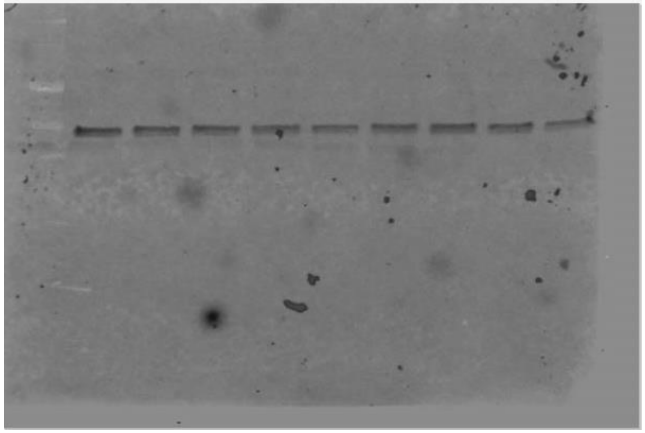

2D-FLAG

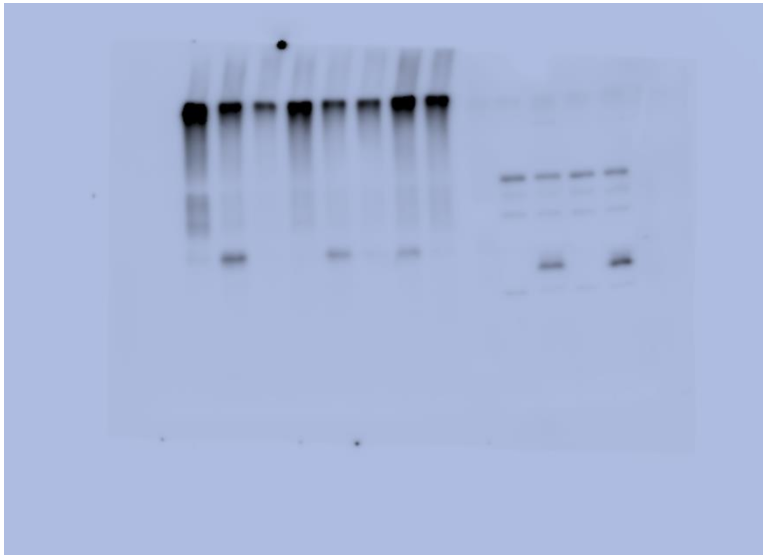

2D-HA

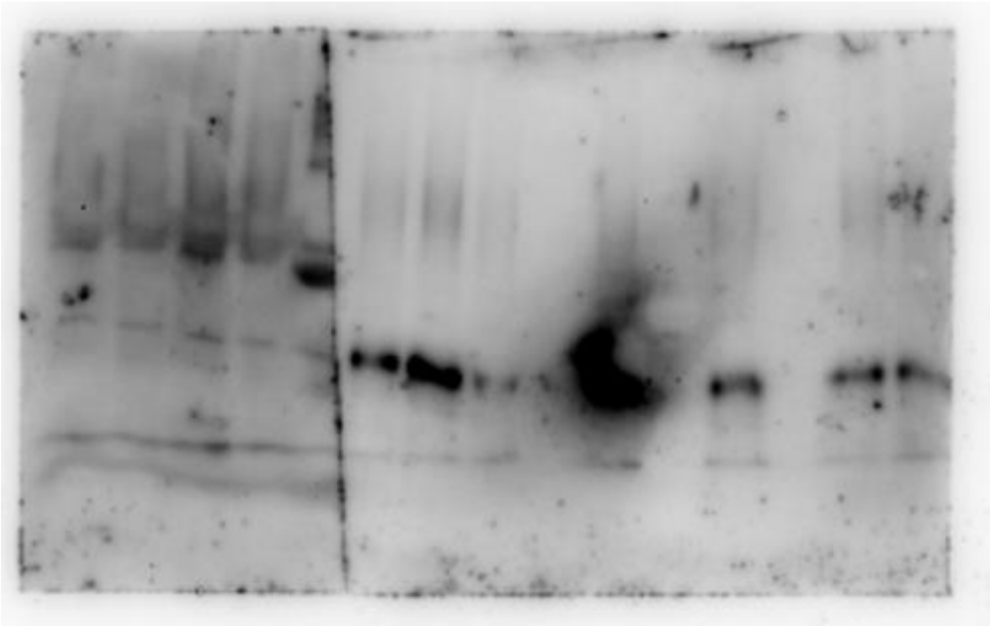

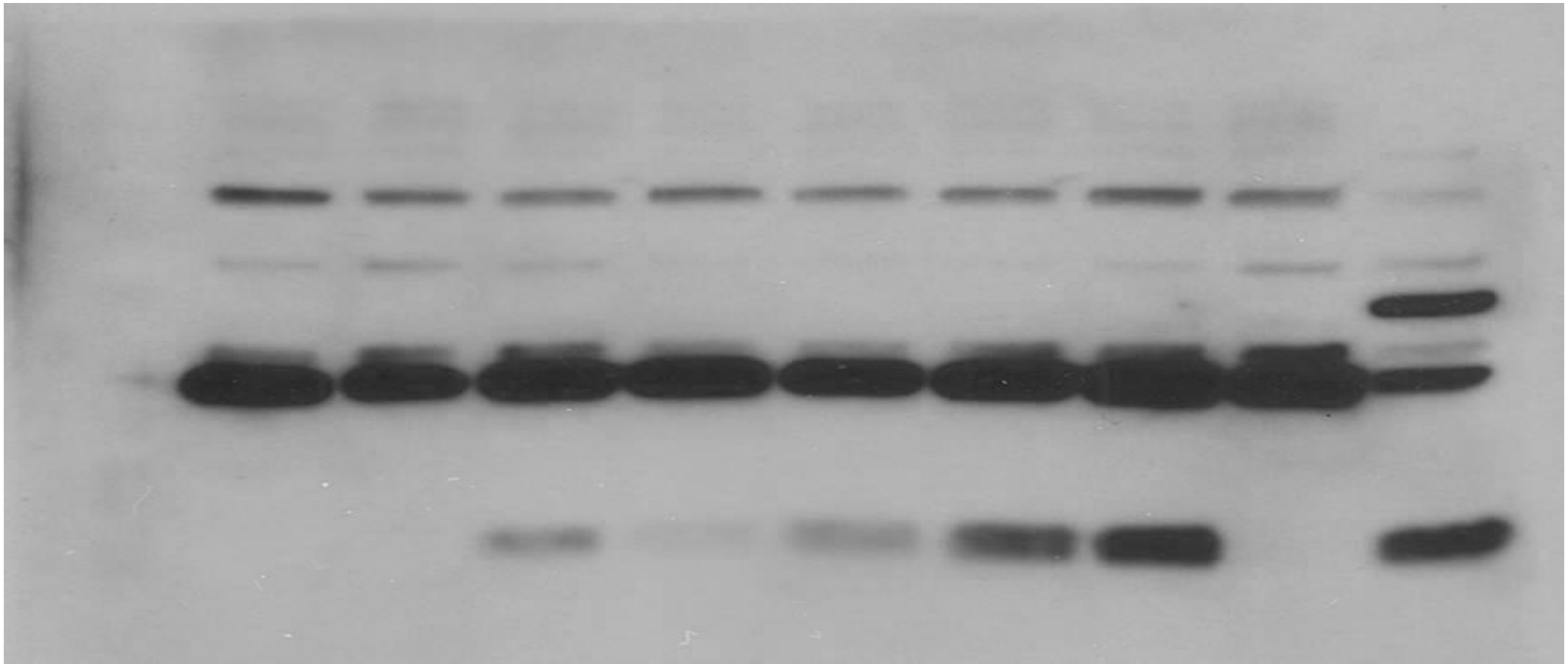

Figure S2
